# Stimulating a future-oriented mindset and goal attainment through a smartphone-based intervention: Study protocol for a randomized controlled trial

**DOI:** 10.1016/j.invent.2022.100509

**Published:** 2022-02-15

**Authors:** Esther C.A. Mertens, Job van der Schalk, Aniek M. Siezenga, Jean-Louis van Gelder

**Affiliations:** a Institute of Education and Child Studies, Leiden University, the Netherlands; b Department of Criminology, Max Planck Institute for the Study of Crime, Security and Law, Germany

**Keywords:** Future self, Future-orientation, Goals, RCT, Smartphone application, Short-term mindsets

## Abstract

**Background:**

Short-term mindsets interfere with the consideration of future consequences and therefore predict negative behaviors. We developed a smartphone-based intervention aiming to increase a future-oriented mindset and personal goal attainment by strengthening future self-identification and stimulating episodic future thinking. The aims of the study are 1) to examine users' experiences with the application and their treatment adherence, 2) to examine the effectiveness of the intervention, and 3) to explore which intervention modules generate the strongest changes in key outcomes.

**Methods:**

First-year university students (*N* = 166) will be randomly assigned to two conditions: 1) the smartphone-based intervention, or 2) a goal-setting control group. The intervention consists of three week-long modules. Data will be collected at the start of the intervention, at weekly intervals during the intervention, immediately after the intervention, and at 3-month follow-up (and at parallel time points for the control group). We will assess users' experiences, application usage data, primary intervention outcomes (e.g., self-defeating behavior, future orientation, future self-identification), and secondary intervention outcomes (e.g., psychosocial wellbeing, self-efficacy).

**Discussion:**

The study will provide information about users' experiences with the application, the intervention's general effectiveness, and which intervention modules show most promise. This information will be used to further develop the application and optimize this novel intervention.

**Trial registration:**

The trial is registered in the Netherlands Trial Register (number: NL9671) on 16 August 2021.

## Highlights

• A smartphone intervention to increase a future-oriented mindset and goal attainment
• Future self-identification as working mechanism in intervention
• Examining users' experiences with the application and intervention effects via an RCT

## 1 Introduction

Short-sighted behaviors, such as substance use, overspending, and unhealthy diets, are known to be generally self-defeating and to generate adverse consequences across a variety of domains (e.g., health, well-being, finance; Hershfield et al., 2011; Rutchick et al., 2018). Such behaviors tend to offer immediate gains, benefitting the ‘present self’, while instilling costs in the longer run, and hence harming the ‘future self’ (Van Gelder et al., 2013, Van Gelder et al., 2022). Conversely, being oriented towards the future stimulates behaviors beneficial to one's psychosocial development and well-being (e.g., Steinberg et al., 2009). To stimulate a future-oriented mindset and personal goal attainment, we developed the smartphone-based intervention ‘FutureU’. The goal of the current study is to examine users' experiences and the intervention's effects in order to further develop the intervention.

### 1.1 Theoretical framework of the intervention

One class of perspectives on intertemporal decision making (i.e., decisions involving tradeoffs between costs and benefits occurring at different timepoints; Frederick et al., 2002) attributes differences in short-sighted versus future-oriented decision making to the discrepancy between the needs and wants of the present self versus those of the future self (Hershfield, 2011, Hershfield, 2018). Short-sighted decisions and behaviors, according to these perspectives, result from a lack of psychological connection with the self in the future. When feeling disconnected from the future self, discounting one's “own” future utility may be no more irrational than discounting the utility of someone else (Frederick et al., 2002). Conversely, a strong sense of psychological connection between the present and the future self is likely to result in decision making favoring the future self by prioritizing the future over the present (Hershfield, 2018).

Previous research has shown that strengthening identification with the future self can stimulate a future-oriented mindset and affect behavior in a variety of domains, including health (Rutchick et al., 2018), delinquency (Van Gelder et al., 2015, Van Gelder et al., 2022), and saving (Hershfield et al., 2011). Most research investigating future self-identification has focused on the effects of exposure (imaginal or through images) to the future self on subsequent behaviors and thinking (e.g., Hershfield et al., 2011; Rutchick et al., 2018). The current intervention combines exposure to the future self with episodic future thinking, which refers to the capacity to imagine or to simulate experiences that might occur in one's personal future (Schacter et al., 2017). According to Suddendorf and Corballis (2007), humans have the capacity to ‘pre-live’ events by mentally projecting themselves forward in time, enabling them to plan for the future. This capacity has been used in interventions addressing lifestyle behaviors, such as food purchases (Hollis-Hansen et al., 2020), eating behaviors (Daniel et al., 2013), and smoking (Stein et al., 2016). The vividness of the imagined future event is an important factor in this process: The more vivid the prospective image is, the stronger the intervention effect (Daniel et al., 2013).

### 1.2 Components of the smartphone-based intervention

Smartphone-based interventions have shown positive effects on a broad range of outcomes, such as depressive symptoms, anxiety symptoms, stress levels, and quality of life (Linardon et al., 2019). Research has started to link these effects to specific components used in applications. For instance, setting goals has been related to positive intervention effects in a randomized factorial trial examining the effectiveness of a smartphone application aimed at increasing physical activity (Fanning et al., 2017), as well as in a systematic review studying characteristics of efficacious smartphone-based interventions aimed at improving lifestyle choices (Schoeppe et al., 2016). Receiving personalized feedback on progress has also been associated with positive effects (Fanning et al., 2017; Schoeppe et al., 2016). Similarly, a meta-analysis examining components of smartphone-based interventions for mental health problems found that push notifications as reminders to use the application, and personalized feedback and supportive messages, were related to stronger intervention effects (Linardon et al., 2019).

### 1.3 FutureU: a smartphone-based intervention

Building on earlier research, FutureU aims to stimulate a future-oriented mindset and personal goal attainment by strengthening people's ability and motivation to consider their future and fostering their identification with their future self. The intervention consists of three modules, each lasting seven days. The first module focuses on future self-identification and personality, the second on temporal distancing and wise reasoning in decision making, and the third on stimulating a growth mindset (Dweck and Yeager, 2019) and goal achievement. By engaging with their future self on a daily basis, we expect participants to gain a more vivid and positive view of their future and to incorporate their future self into their own identity. This positive vivid image of the future self and its integration into one's identity is expected to foster future-oriented mindset and behaviors (e.g., future orientation, reduced self-defeating behaviors, psychosocial well being), and personal goal attainment (Nurra and Oyserman, 2018; Verplanken et al., 2008).

The intervention includes components that have been related to positive intervention effects in prior interventions. Goal setting, personalized feedback, and supportive messages are implemented throughout the intervention. Participants set goals both at the start of the intervention and on a weekly basis during the intervention. Personalized feedback and supportive messages, which are partially based on participants' responses in the application, are communicated via the future self, such as the advice they formulate for their present self from the perspective of their future self. Although the feedback and support messages are not provided by a therapist, automatically generated personalized support has been shown to generate equivalent effects (Linardon et al., 2019).

We use technology to create a persuasive three-dimensional visual analog, an ‘avatar’, of participants' future self, and create the suggestion of interaction with this future self. Moreover, we developed an interaction within the application, a ‘time travel portal’, intended to facilitate mental time travel and alternating between taking the perspective of the future self and that of the present self. Furthermore, the intervention is standardized, ensuring intervention fidelity, and stimulates engagement with the application through the use of daily push notifications.

### 1.4 The present study

The current study has three aims. First, we will examine users' experiences with the application and intervention adherence. This provides important information for the further development of the FutureU smartphone application, as high levels of acceptability and usability increases users' tendencies to apply the recommended intervention techniques (Sekhon et al., 2017). Second, we will analyze the overall effectiveness of the intervention. This will provide an indication about the extent to which the intervention is able to stimulate a future-oriented mindset, increase identification with the future self, promote beneficial behaviors, reduce self-defeating behavior, and foster personal goal attainment. Third, we will explore participants' change on several outcomes during the intervention to study the effectiveness of each module. We specifically focus on concepts in which changes can be measured over the course of a week, such as self-defeating behaviors (e.g., missing classes or work, overspending, procrastinating) and psychosocial wellbeing. Studying change on key outcomes after each module allows for determining which modules carry most promise for establishing change. This information provides detailed insights into how the intervention can be developed further to improve effectiveness and carries theoretical relevance (Fanning et al., 2017).

Given that interventions aimed at changing behavior are best implemented during a transformational life event and change of context (Dai et al., 2014; Verplanken et al., 2008), the target population of the current study is first-year university students. For many of these students transitioning from secondary school to university involves moving to a new city, living by themselves, being separated from family and close others, and/or taking on new roles. During transformational life events, people are more inclined to take a ‘big-picture’ view of their lives, which may motivate behavioral changes (Dai et al., 2014). Additionally, a change of context can alter choices and decisions based on contextual information in order to adapt to the new situation (Verplanken et al., 2008).

We hypothesize that participants experience the smartphone application as user friendly and engaging, and use it multiple times per week. Furthermore, we hypothesize that the intervention is effective in increasing students' future self-identification, future-oriented mindset and behaviors (e.g., increasing future-orientation, reducing self-defeating behavior), and personal goal attainment. Regarding the effectiveness of the separate intervention modules, we hypothesize to see positive intervention effects after each module, given that all modules are based on theoretical mechanisms of change. It is important to note, however, that to the best of our knowledge, this is the first time these theories of change are combined and incorporated in a smartphone application, which, in turn, may affect the effectiveness of these theorized mechanisms.

## 2 Methods

### 2.1 Design

The effectiveness of the intervention will be examined by means of a Randomized Controlled Trial (RCT) with two conditions: 1) a smartphone-based intervention condition, and 2) an active control condition. In the smartphone condition, participants receive the intervention over a period of three weeks. In the control condition, participants actively set goals, but will receive no further intervention.

All participants start with an individual intake session with a member of the research team in the lab at the university. During this session participants provide active informed consent, set goals for the coming month (i.e., monthly goal) and the year (i.e., yearly goal), and complete a series of questionnaires (baseline assessment). In addition, participants in the smartphone condition will take a photo of their face (a ‘selfie’) for avatar creation and install the application on their phone.

**Fig. 1:**
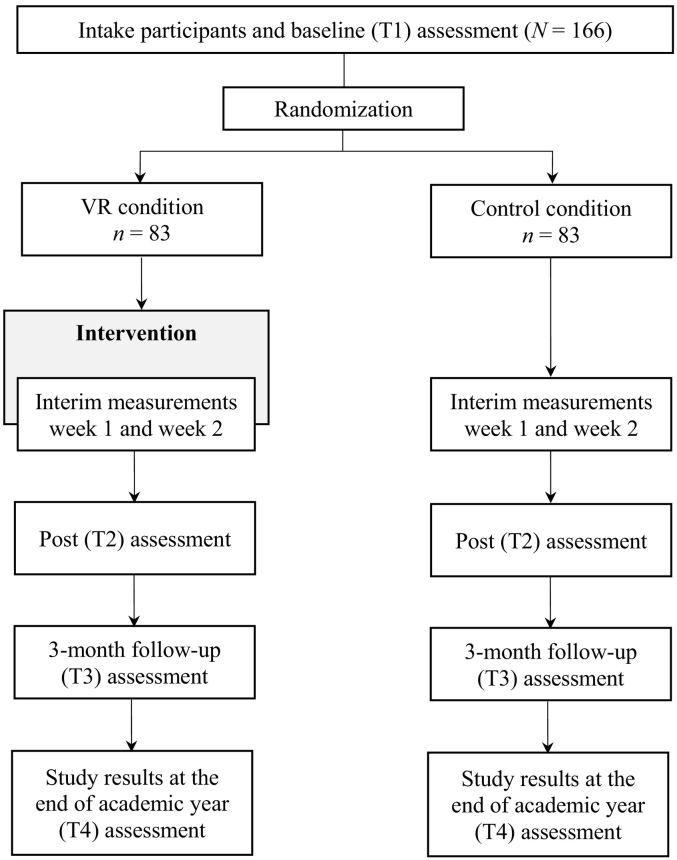
Study flow chart.

Assessments will be conducted prior, during, and immediately after the intervention, and at 3-month follow-up. Participants in the control condition will complete the assessments at parallel points in time. The outcomes will be assessed using online questionnaires (using Qualtrics software) completed by the participants. Academic results will be requested from the university at the end of the academic year after participants' consent. The interim assessments (one week and two weeks after baseline assessment) concern a subset of the outcomes. In exchange for participation, participants receive €25,- or 6 course credits after completion of the post-measurement, and an additional €10,- for completion of the 3-month follow-up (see Fig. 1 for the study flow chart).

Ethical approval for this study was obtained from the independent Ethics Board of the Institute of Education and Child Studies at Leiden University (ECPW2021-320).

### 2.2 Participants and power calculation

Participants are first-year students enrolled in a university in the Netherlands who own an Android or iPhone smartphone. Sample size was determined by an a-priori power analysis with G*Power. Estimation of effect size is based on Tanner-Smith et al. (2018) who examined mean effect size distributions for universal interventions in a review of meta-analyses (Cohen's *d* ranging from 0.23–0.58). Assuming a significance level of *p* < .05, a medium effect size of Cohen's *d* = 0.50, two conditions, and one covariate, a sample size of 128 participants is estimated to achieve a power of 80%. Considering a drop-out rate of 20%, we aim to include 83 participants per condition, resulting in a total sample of 166 participants.

### 2.3 Recruitment and randomization

Participants will be recruited via the university's official communication channels (e.g., university website, social media). Additionally, flyers will be distributed in university buildings. Furthermore, student associations will be requested to distribute the study advertisement among their members. Finally, the study will be advertised during the university's introduction week for incoming students, and announced at lectures for first-year students.

Interested students can schedule an appointment for the intake session. At the beginning of the session, students are screened for eligibility. Eligible students are randomly assigned to one of the two conditions on a 1:1 ratio, using block randomization with blocks of 6 (i.e., within a block 3 participants are allocated to each condition), and proceed with the intake session.

### 2.4 Blinding

It is not possible to blind participants or researchers to conditions as all will know whether they will be using the smartphone application or not. Participants are, however, unaware of the content of the application or the study's hypotheses.

### 2.5 Conditions

#### 2.5.1 Intervention

During the intake, participants formulate a monthly- and a yearly goal. The goals can be related to each other, but this is not a requirement. Goal-setting is guided by the researcher to ensure that the goals are specific, measurable, and challenging but attainable, as goals with these characteristics are most likely to have a positive effect on performance (Van Lent and Souverijn, 2020). To this end, goals are determined using the SMART-goal model and Zimmerman's criteria (Ogbeiwi, 2021). Participants also formulate a weekly goal that will facilitate attainment of their monthly goal. During the intake participants independently formulate the first weekly goal, with a reminder of the researcher to consider the SMART-goal model. At the end of each week, participants indicate to which extent they have worked on, and reached, their goal of that week and independently formulate a new weekly goal.

**Fig. 2:**
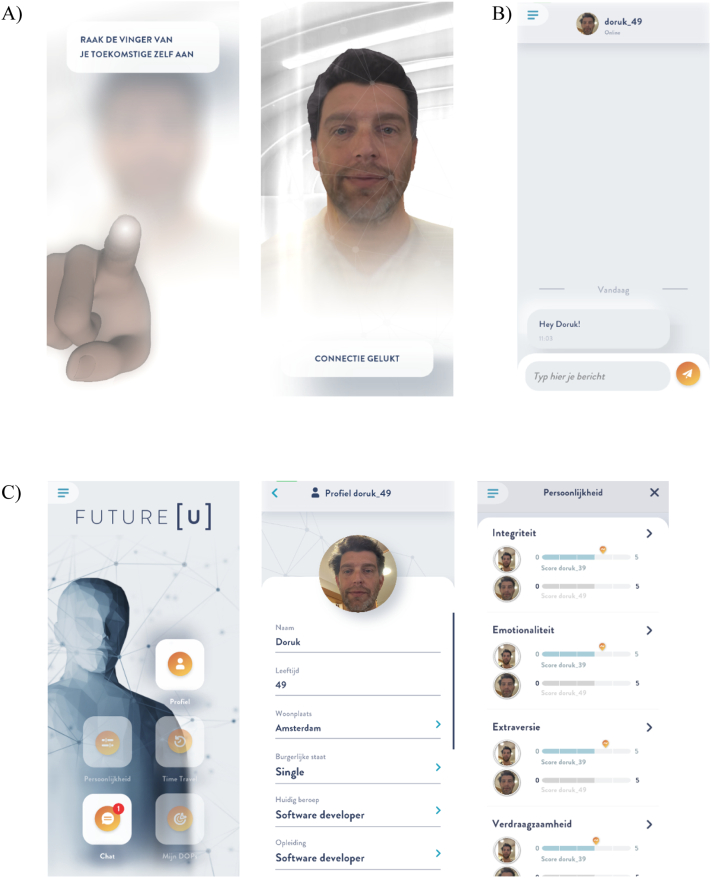
FutureU application screenshots of A) connection, B) chat, and C) homescreen and menus.

Prior to this RCT, the first version of the FutureU application was extensively user-tested. Semi-structured interviews and focus groups were held among users belonging to the target population as well as experts (i.e., application developers, researchers, and a therapist with experience in smartphone-based interventions). User feedback was processed and used to further improve the application. An overview of module content and core features is provided in Table 1. For screenshots of the application, see Fig. 2.

**Table 1:** Modules of the intervention.

Week	Module	Aim	Theory	Core features
1	Future self and personality	Stimulating vividness, familiarity and identification with the future self and learn about their own and their future selves' personality	• Exposure to and vividness of the future self increases future orientation (McMichael et al., 2021). • Incremental personality theory: The belief that personality can change over time can reduce problematic behaviors (Yeager, 2017). • People's willingness to change on personality traits in socially desirable ways increases after feedback on their current trait levels (Thielmann and De Vries, 2021).	• Complete personal profile of the future self (e.g., work experience, skills, accomplishments) • Current scores on personality traits with an indication of norm scores • Psychoeducation that personality can change over time • Set scores of personality traits of future self
2	Future self perspective	Practice with distanced perspective taking on problems to make future-oriented choices and increase self-insight with the potential to adjust attitudes and behaviors in favor of the future self	• People make more future-oriented choices: 1) for others (i.e., Solomon's paradox; Grossmann and Kross, 2014) 2) when they have a vivid perception of the future self (McMichael et al., 2021) 3) when they can psychologically or temporally distance themselves from the situation (i.e., Construal level theory; Trope and Liberman, 2003). • Wise reasoning is enhanced with third-person self-reflection (Grossmann et al., 2021)	• Psychoeducation that people make more future-oriented choices when they distance themselves from the situation, and when they think about the long-term consequences • Time travel portal to take future self perspective for giving compliment, advice, and motivation • Participants address themselves in the third-person • Spoken interaction with playback of the recorded messages
3	Goal setting and achievement	Educate about a growth mindset to stimulate goal setting and practice Mental Contrasting and Implementation Intentions to foster goal achievement	• Growth mindset: The belief that people's abilities can develop over time. This mindset aids engagement in thoughts and behaviors to work towards goals (Dweck and Yeager, 2019). • Mental Contrasting and Implementation Intentions (Oettingen and Gollwitzer, 2010): Method in which the desired future is contrasted with the current reality and then reflected upon obstacles in the way of attaining the desired future. Subsequently, a plan is formulated to implement behaviors to overcome obstacles, i.e., implementation intentions, in the format: If situation X, then I will do Y.	• Psychoeducation that abilities can develop over time • Video-clip explaining Mental Contrasting and Implementation Intentions • Practice with Mental Contrasting and Implementation Intentions to work towards goals via filling in a scheme

The main recurrent feature of the application is the interaction with the future self via a chat function (Fig. 2A). During the entire intervention period (i.e., 21 days), participants receive a daily push notification stating that there is a message from their future self. Every other day, participants receive an additional push notification with a general comment of their future self. Every time after opening the application, participants first ‘connect’ with their future self by touching the (virtual) finger of their future self on the blurred screen of their phone. This generates a pulse and unblurs the screen with the avatar of the future self becoming visible (see Fig. 2B). The purpose of this ‘connection mechanic’ is to establish a kinetic connection with the future self and to present participants with an affiliation smile as positive social reinforcement for using the application (Martin et al., 2017). After connection is established, participants enter the chat to interact with their future self as they engage in a scripted conversation (see Fig. 2C). In the chat, the future self provides psychoeducation (both in text and in video-clips), asks questions relating to the participants' perceived future, or provides instructions for the (theory-driven) interaction of that day. The future self also sends images and emojis, and makes jokes to keep the interaction fun. Daily interactions in the application last approximately 5 min or less, as the application favors frequency of contact over length.

#### 2.5.2 Control

Participants in the control condition set a monthly- and a yearly goal during the intake, following the same procedure as participants in the intervention condition (see Section 2.5.1 Intervention). Furthermore, participants set a weekly goal that helps them to work towards their monthly goal. As in the intervention condition, the first weekly goal is set independently during the intake with a reminder of the researcher to keep the SMART-goal model in mind when formulating the goal. The other weekly goals are formulated independently by the participant at the end of the weekly questionnaires.

In contrast to the intervention condition, participants do not receive further support for working towards their goals. As the mere act of setting specific, measurable, and challenging goals has been shown to increase positive outcomes (e.g., Van Lent and Souverijn, 2020), this active control condition allows us to examine intervention effects over and above the potential effects of setting goals.

### 2.6 Measures/instruments

An overview of the concepts, instruments, and measures at each time point is presented in Table 2.

**Table 2:** Overview concepts, instruments and assessment time points administered.

Concept	Instrument	#Items	T1	Int.	T2	T3	T4
Future orientation	Future Orientation Scale	15	x		x	x	
Self-defeating behavior	Self-defeating behavior list based on Van Gelder et al. (2015)	16	x	x	x	x	
Goal commitment	Goal Commitment Questionnaire	7	x		x	x	
Weekly goal achievement	Self-developed	3		x	x		
Future self-identification	Based on Hershfield et al. (2009) and Van Gelder et al. (2015)	8	x	x	x	x	
Academic results^a^	University records	–					x
Impulsiveness	Barratt Impulsivity Scale 15 item version	15	x		x	x	
Engagement with application^b^	Smartphone application usage data	–			x		
Smartphone application experiences^b^	Based on mHealth App Usability Questionnaire and the Mobile App Rating Scale	27			x		
Personality	HEXACO-PIR-R100	100	x				
Psychosocial wellbeing	Warwick-Edinburgh Mental Wellbeing scale	14	x	x^c^	x	x	
Self-efficacy	General Self-efficacy Questionnaire	10	x		x	x	
Self-esteem	Rosenberg Self-esteem Scale	10	x		x	x	

a University records will be requested at the end of the academic year (T4).

b Only assessed in the smartphone-based intervention condition.

c The 7-item short version is used for the interim assessments. Int = interim assessments.

#### 2.6.1 Primary outcomes

Future orientation will be assessed with the Future Orientation Scale (Steinberg et al., 2009), which measures time perspective, anticipation of future consequences, and planning ahead. The scale consists of 15 items (e.g., “Some people spend very little time thinking about how things might be in the future, but other people spend a lot of time thinking about how things might be in the future.”; α = 0.80).

The self-defeating behavior measure, indicating behaviors with immediate gains and long-term costs, is based on the measure used in Van Gelder et al. (2015). The scale consists of 16 items representing different self-defeating behaviors (e.g., “How often in the past week have you used drugs?”).

The extent to which participants are committed to their year goal will be assessed with the Goal Commitment Questionnaire (Hollenbeck et al., 1989) consisting of 7 items (e.g., “I think this goal is a good goal to shoot for.”; α = 0.71).

Weekly goal achievement will be measured after each week of the intervention, or at parallel time points in the control condition, with 3 items developed for this study: “I often thought about my weekly goal.”, “I have worked towards my weekly goal.”, and “I have achieved my weekly goal.”.

Future self-identification indicates the degree to which people have a clear image of their future self and can identify with their future self. We will assess the vividness with which people can imagine their future self (5 items, e.g., “I find it easy to imagine myself 10-years in the future.”; based on Van Gelder et al., 2015), positive valence towards their future self (1 item, “Indicate how you feel when you think about the future.” answer categories ranging from negative to positive; based on Hershfield et al., 2009), and relatedness with their future self (2 items, e.g., “How connected do you feel with your future self?”; based on Hershfield et al., 2009).

Academic results at the end of the academic year will by requested from university, after student consent.

Impulsiveness is assessed with the Barratt Impulsiveness Scale short form measuring non-planning, motor impulsivity, and attentional impulsivity (Spinella, 2007). The short form consists of 15 items (e.g., “I do things without thinking.”; α = 0.79).

In the smartphone condition, we will additionally assess engagement with the application through usage data and users' experiences with the application immediately after the intervention through a questionnaire. User experience measures (27 items in total) include ease of use (e.g., “The app was easy to use.”), satisfaction (“I am satisfied with the app.”), and acceptability (e.g., “I would use the app again.”) of the application. They are based on the subscale Ease of use and satisfaction of the mHealth App Usability Questionnaire (Zhou et al., 2019) and the subscale Functionality of the Mobile App Rating Scale (Stoyanov et al., 2015).

#### 2.6.2 Secondary outcomes

Personality will be assessed at baseline with the 100-item HEXACO-PI-R (Lee and Asthon, 2018) which measures 6 dimensions: Honesty-Humility, Emotionality, Extraversion, Agreeableness, Conscientiousness, and Openness to Experience. Each dimension is assessed with 16 items (e.g., “I often check my work over repeatedly to find any mistakes.”, “I am energetic nearly all the time.”, “Having a lot of money is not especially important to me.”), with the interstitial facet Altruism measured with 4 items. The six HEXACO dimensions have shown high reliability (range α = 0.82–0.89). Measuring personality enables personalized feedback in the first module of the application (see also Table 1).

Psychosocial wellbeing will be measured with the Warwick-Edinburgh Mental Wellbeing scale (Tennant et al., 2007), which is a broad measure of positive mental health. The questionnaire consists of 14 items (e.g., “Last week, I have been feeling relaxed.”; α = 0.84). The short 7-items version will be used for the interim measurements (Ng Fat et al., 2017).

Self-efficacy, referring to one's sense of competence to effectively deal with life stressors, will be assessed with the General Self-efficacy Questionnaire (Schwarzer and Jerusalem, 1995). The questionnaire consists of 10 items (e.g., “I can always manage to solve difficult problems if I try hard enough.”; range α = 0.75–0.91).

Self-esteem will be assessed with the Rosenberg Self-esteem Scale (Rosenberg, 1965) measuring people's global evaluation of themselves. The scale consists of 10 items (e.g., “On the whole, I am satisfied with myself.”; α = 0.86).

### 2.7 Data management

All members of the research team have signed a confidentiality statement, are familiar with procedure to manage and store data (in line with the guidelines and procedures of the Ethics Board of the Institute of Education and Child Studies at Leiden University (ECWP2021-320)), and have access to the data. Data is collected online and will be stored on secured servers of Leiden University that are backed up regularly. To monitor data quality, attention checks will be incorporated in the questionnaires.

### 2.8 Statistical analyses

Data will be analyzed on the basis of an intention-to-treat principle, meaning that participants will be included in the analyses regardless of whether they received the (complete) intervention or not. Depending on the analytical model, missing data will be handled either with Multiple Imputation (MI) or with Full Information Maximum Likelihood (FIML). Conditions will be compared at baseline to examine initial differences on age and gender. In case of differences between conditions, these variables will be controlled for in subsequent analyses.

Users' experiences and adherence to the intervention (aim 1) will be examined through the examination of usage data and user experience measures. The overall effectiveness of the intervention (aim 2) will be examined by comparing conditions on the primary and secondary outcomes (except for personality) using Analysis of Covariance (ANCOVA), with the baseline measure of the relevant outcome as covariate, and Latent Growth Curve (LGC) modeling. This enables us to examine differences between conditions immediately after the intervention and whether trajectories of change differ between conditions. To examine participants' change after each intervention module (aim 3), we will conduct repeated measures ANOVAs on the outcomes that are measured on a weekly basis, i.e., self-defeating behavior, future self-identification, and psychosocial wellbeing.

## 3 Discussion

The FutureU smartphone intervention aims to increase people's future orientation and personal goal attainment by strengthening their future self-identification. FutureU combines theoretical mechanisms of change capitalizing on affordances offered by smartphone technology. In the present RCT, both users' experiences with the application and intervention effects will be assessed.

Although the use of smartphone applications brings multiple advantages, it also introduces challenges. One important challenge regards the risk of participant drop out. Not only is the risk of drop-out generally high in smartphone-based interventions (Schoeppe et al., 2016), our intervention also requires a relatively high level of commitment from participants: Daily participation in an intervention for 3 weeks and multiple measurement points throughout the study (including a 3-month follow-up). The inclusion of multiple measurement points during and after the intervention is an essential aspect of the study design to test its effectiveness, but also requires time, effort, and engagement from participants. To decrease participant burden, we limited interaction time to a maximum of 5 min. Furthermore, we made an effort to make the intervention engaging (e.g., by using animations, funny images) and spent ample time and effort on design and user-interaction. Involving both the target population and experts in an extended user-test provided us with feedback and suggestions to improve the intervention. Finally, participants receive compensation (course credits and/or monetary rewards) for their participation to further reduce drop-out.

In conclusion, the current study examines users' experiences and intervention effects of a novel smartphone-based intervention. The knowledge gained from this study will be used for further development of the content and features of the intervention. Although the current study targets university students, the intervention has the potential to be relevant for other populations as well. Given that the extent to which people have a clear and vivid image of their future is related to a wide array of domains such as health, delinquency, and saving (Hershfield, 2011; Rutchick, 2018; Van Gelder et al., 2015, Van Gelder et al., 2022), an intervention that can strengthen people's future self-identification has the potential to indirectly stimulate positive outcomes within these domains. This potentially broad impact underscores the relevance of our smartphone-based intervention and the importance of its optimization.

## Declarations

### Ethics approval and consent to participate

Ethical approval for conducting this RCT is obtained from the independent Ethics Board of the Institute of Education and Child Studies at Leiden University (ECPW2021–320). Students will provide active informed consent for their participation in the study.

## Funding

The study is financially supported by the ERC Consolidator Grant (Grant Number 772911-CRIMETIME). The funder had no role in study design, data collection and analysis, preparation of the manuscript, and decision to publish.

## CRediT authorship contribution statement

JLvG obtained funding for the study. All authors contributed to the conceptualization of the intervention and study design. EM wrote the draft of the manuscript. All authors critically revised the manuscript. All authors read and approved the final manuscript.

## Declaration of competing interest

The authors declare that they have no known competing financial interests or personal relationships that could have appeared to influence the work reported in this paper.
